# House value as an individual socioeconomic indicator for breast cancer survival and late-stage diagnosis: a population-based cohort study from Northern Ireland

**DOI:** 10.1007/s10549-026-07947-z

**Published:** 2026-03-21

**Authors:** Sarah M. Baxter, Charlene M. McShane, Stuart A. McIntosh, Damien Bennett, Meenakshi Sharma, Lynne Lohfeld, Daniel R. S. Middleton, Gerard Savage, Deirdre Fitzpatrick, Ann McBrien, David McCallion, Anna Gavin, Chris R. Cardwell

**Affiliations:** 1 Centre for Public Health, Queen’s University Belfast, Belfast, Northern Ireland, UK; 2 The Patrick G Johnston Centre for Cancer Research, Queen’s University Belfast, Belfast, Northern Ireland, UK; 3 Breast Surgery Department, Belfast City Hospital, Belfast Health and Social Care Trust, Belfast, Northern Ireland, UK; 4 Northern Ireland Cancer Registry, Belfast, UK; 5 Patient Representative, Northern Ireland, UK; 6 Patient Representative, England, UK

**Keywords:** Breast cancer, Survival, Stage, Socioeconomic position, House value, Property value

## Abstract

**Purpose:**

Socioeconomic inequalities in breast cancer survival persist in the UK. Area-based deprivation measures may underestimate socioeconomic effects by assigning average deprivation levels to all area inhabitants. This study investigated associations between house value (individual-level) and area-based deprivation with breast cancer outcomes in Northern Ireland.

**Methods:**

Women diagnosed with breast cancer from 2011 to 2021 were identified using the Northern Ireland Cancer Registry. House value was determined from Valuation and Lands Agency property valuation data, and area-based deprivation from the Northern Ireland Multiple Deprivation Measure. The primary outcome was breast cancer-specific mortality. Secondary outcomes were stage at diagnosis and all-cause mortality. Cox regression models calculated adjusted hazard ratios (HR) and 95% confidence intervals (95% CIs) for mortality by house value category and deprivation, adjusting for confounders.

**Results:**

Among 12,766 women with breast cancer, women in the lowest versus highest house value category had a 60% increase in mortality (adjusted HR = 1.60 95% CI 1.34, 1.92; per 20 percentile decrease adjusted HR = 1.12 95% CI 1.08, 1.16) and were more likely diagnosed with stage 4 disease (7.5% versus 4.1%; *P* < 0.001). Women living in the most versus least deprived areas had a 26% increase in mortality (adjusted HR = 1.26 95% CI 1.08, 1.47; per 20 percentile decrease adjusted HR = 1.05 95% CI 1.01, 1.08) but had no difference in stage 4 disease (5.9% vs. 5.0%; *P* = 0.157).

**Conclusion:**

Individual-level house value demonstrated stronger associations with breast cancer outcomes than area-level deprivation, suggesting it may serve as a more sensitive indicator for monitoring health inequalities in cancer.

**Supplementary Information:**

The online version contains supplementary material available at 10.1007/s10549-026-07947-z.

## Introduction

Breast cancer is the most diagnosed cancer among women worldwide, with over 2.3 million cases in 2022 [1]. Although survival has improved in recent decades [2, 3], patients from lower socioeconomic positions (SEP) have demonstrated poorer survival, both in the UK [4, 5] and internationally [6–8]. Despite efforts to reduce socioeconomic inequalities in cancer outcomes within the UK, disparities persist [9–11].

Most research on socioeconomic inequalities in breast cancer mortality has focused on area-based deprivation measures [5], as cancer typically lacks individual-level SEP measures. Area-level deprivation measures allocate individuals to administrative geographical areas and aggregate indicators across these areas. Advantages include publicly available data covering entire populations and capture different deprivation domains.

However, area-level measures have recognised limitations. They assign individuals the average deprivation level of their area, which reduces the scale of inequalities towards the null [12]. Additionally, area-based and individual-level measures often classify patients, including cancer patients [13], differently. For instance, affluent individuals living in deprived areas are classified according to their area-level rather than individual SEP, and vice versa, with misclassification increasing as geographies become larger [12].

Investigating individual-level SEP is important to better understand the mechanisms underlying health inequalities [13]. Previous studies have shown associations between lower individual-level SEP (measured by household income and education) and later stage at diagnosis [14, 15], although the association with breast cancer survival is not consistent [16]. House value, reflecting both economic resources and neighbourhood context [17, 18], has been proposed as a novel individual-level SEP measure, particularly for older populations who may have retired, making traditional occupation-based or income-based SEP measures less relevant [17]. In the UK context, where homeownership rates are high [19] and property wealth constitutes a substantial portion of household assets [17, 20, 21], house value may be especially relevant.

Despite this, few studies have previously investigated house value and mortality in breast cancer patients [22, 23]. In Northern Ireland (NI), the house valuation for all domestic properties is publicly available [17], making this a potentially useful population-level SEP measure. Consequently, we examined the association between individual-level house value and area-based deprivation with breast cancer survival and stage at diagnosis in a large cohort of breast cancer patients from NI.

## Methods

### Data sources

The study utilised data from the Northern Ireland Cancer Registry (NICR), which captures comprehensive information on all cancer patients including age, date of diagnosis, tumour site, tumour stage and grade, as well as death registrations (from the General Registrar Office) with estimated completeness exceeding 99% [24, 25]. Patient residential addresses at cancer diagnosis were obtained from the NI Health Card registration system and linked to property valuations from the Valuation and Lands Agency Northern Ireland-wide government property valuation exercise using unique property identifiers.

Prescription medication data were taken from the Northern Ireland electronic prescribing dataset (NIEPD), maintained by the Health and Social Care Business Services Organisation, which captures all NHS prescriptions dispensed by community pharmacists in NI. All datasets were linked using a unique patient identifier (NI Health and Care Number) by HSCNI Honest Broker Service [26]. All analyses were conducted within the Honest Broker Service secure environment.

### Cohort

We identified all women newly diagnosed with invasive breast cancer (ICD-10 code C50) between January 2011 and December 2021, excluding those with a previous cancer diagnosis (except non-melanoma skin cancer).

### Outcome

The primary outcome was breast cancer-specific mortality, defined as death with breast cancer (ICD-10 code C50) as the underlying cause of death. Secondary outcomes were stage at diagnosis and all-cause mortality.

### Exposure

The exposure variables of interest were house value of patients’ residence at time of breast cancer diagnosis (household/individual-level SEP) and the 2017 Northern Ireland Multiple Deprivation Measure [27] (NIMDM) classification (area-level classification).

To determine house value, patient addresses at diagnosis were obtained from the Northern Ireland Health Card registration system [28]. Each address was matched to its Unique Property Reference Number (UPRN) using the Pointer address database maintained by Land and Property Services. The rateable house value, which is still used in 2025 to determine local tax levels, was obtained and was based upon a 2007 valuation exercise undertaken by the former Valuation and Lands Agency in which all domestic properties were valued according to their estimated open market value as of 2005. Properties built after 2005 were assigned values equivalent to comparable pre-2005 properties [29]. House values were available in eight categories (in £1000 s): < 75, 75–99, 100–124, 125–199, 200–249, 250–299, 300–399, and ≥ 400. For this analysis, house values were classified into the following five categories (in £1000 s): < 75, 75–99, 100–124, 125–199, ≥ 200. Although house values were based on 2005 valuations, this approach should not introduce bias provided the relative ranking of property values across different areas remained stable over the study period [30]. House value likely serves as a reasonable SEP marker for renters, as private renters typically rent in areas that reflect their socioeconomic position and social housing tenants are likely to reside in lower-value properties.

The NIMDM was determined for each patient’s postcode at diagnosis, based upon Super Output Areas (geographic units of approximately 2100 people), capturing seven domains of deprivation: income, employment, health, education, access to services, living environment and crime, and was analysed in fifths.

### Covariates

The NICR provided information on year of diagnosis, age at diagnosis, tumour stage and grade, and pathway to diagnosis (screen detected, red flag referral, death certificate only and other [e.g. emergency presentation]). Treatment data within the first year of diagnosis included surgery (mastectomy or breast-conservation), systemic therapy (including chemotherapy) and radiotherapy. Use of endocrine treatments (tamoxifen or aromatase inhibitors) was determined from dispensed medications recorded in the NIEPD.

Pre-existing comorbidities were identified from hospital admissions data up to five years prior to breast cancer diagnosis. The following conditions from the Charlson Comorbidity Index were identified based on ICD-10 codes as a cause of hospital admission from Patient Administration System, using previously used code lists [31]. The conditions included myocardial infarction, congestive heart disease, peripheral vascular disease, cerebrovascular disease, chronic pulmonary disease, dementia, liver disease, peptic ulcer disease, diabetes and chronic kidney disease.

### Statistical analysis

First, characteristics of patients with breast cancer were determined by house value.

Cox regression analysis was used to examine associations between SEP measures and breast cancer-specific mortality. Follow-up was censored at death from other causes, date of emigration and date of complete mortality records (31st March 2023). Kaplan–Meier curves were plotted to show survival by house value. Three levels of adjustment were applied (1) age at diagnosis, year of diagnosis, individual Charlson comorbidities; (2) additionally including area deprivation; and (3) additionally including tumour stage and grade. The analysis was conducted by house value in categories and per twenty percentile decrease in house value. For the percentile analysis, each house value category was assigned its midpoint percentile value based on the distribution of house values in the breast cancer cohort. Analyses were repeated with area deprivation as the exposure and all-cause mortality as the outcome.

The main analysis used a complete case approach where we excluded patients with missing house value or deprivation. However, we assessed the impact of missing data by conducting two further sensitivity analyses, including a missing category for house value and deprivation, and using multiple imputation for missing house value and deprivation. House value and deprivation (in fifths) were imputed, using chained equations, in 10 datasets using ordinal logistic regression models with cancer-specific death status, cumulative hazard and age at diagnosis, year of diagnosis and Charlson comorbidities in imputation models [32], and results were combined using Rubin’s rules [33].

Logistic regression analysis was used to examine associations between SEP measures and later-stage disease at diagnosis. First, we compared stage 4 disease with stage 1 to 3 disease at diagnosis. Models were fitted separately for house value and area deprivation as exposures. Adjusted analysis included age at diagnosis, year of diagnosis and individual Charlson comorbidities, with area deprivation additionally included in house value models. In sensitivity analysis, we examined stage 4 or stage unknown disease at diagnosis versus stage 1 to 3 disease at diagnosis, stage 3 to 4 versus stage 1 to 2, and stage 3 or 4 or stage unknown versus stage 1. Again, we utilised a complete case approach (those with either a missing house value or deprivation were excluded). All analyses were conducted using STATA 18 (StataCorp, Texas, USA).

## Results

The full cohort included 13,846 women with breast cancer. Overall, 976 (7%) women had a missing house value and 104 (1%) had a missing deprivation score, leaving 12,766 women in the main analysis with complete records for house value and deprivation.

### Characteristics of patients

Characteristics of patients by house value (Table 1) demonstrated clear socioeconomic gradients across several characteristics. Compared with patients living in the most valuable properties, those in the least valuable properties were slightly older, had slightly higher stage, were more likely to be detected via red flag referral, and were less likely to have radiotherapy, systemic therapy and prescribed tamoxifen. Clear differences in area-based deprivation by house value were evident, with only 39% of breast cancer patients in the least valuable properties living in the most deprived areas, while only 45% of patients in the most valuable properties living in the least deprived areas. Other characteristics were similar across house value categories.

**Table 1:** Characteristics of breast cancer patients by house value

Characteristics	House value (per £1000)				
	0–74	75–99	100–124	125–199	> 200
*n*	2483	2608	2118	3722	1835
Year: 2010–13	669 (27%)	695 (27%)	528 (25%)	901 (24%)	432 (24%)
2014–16	647 (26%)	658 (25%)	583 (28%)	1058 (28%)	495 (27%)
2017–19	674 (27%)	759 (29%)	608 (29%)	1076 (29%)	556 (30%)
2020–22	493 (20%)	496 (19%)	399 (19%)	687 (18%)	352 (19%)
Age: < 50	436 (18%)	536 (21%)	478 (23%)	766 (21%)	475 (26%)
50- < 60	530 (21%)	646 (25%)	544 (26%)	985 (26%)	525 (29%)
60- < 70	638 (26%)	635 (24%)	516 (24%)	945 (25%)	440 (24%)
70- < 80	492 (20%)	467 (18%)	358 (17%)	623 (17%)	250 (14%)
80 +	387 (16%)	324 (12%)	222 (10%)	403 (11%)	145 (8%)
Deprivation (in fifths):					
1 st (Most deprived)	964 (39%)	673 (26%)	250 (12%)	219 (6%)	45 (2%)
2nd fifth	673 (27%)	642 (25%)	436 (21%)	605 (16%)	183 (10%)
3rd fifth	474 (19%)	568 (22%)	453 (21%)	733 (20%)	275 (15%)
4th fifth	283 (11%)	452 (17%)	535 (25%)	1003 (27%)	507 (28%)
5th (least deprived)	89 (4%)	273 (10%)	444 (21%)	1162 (31%)	825 (45%)
Stage: 1	936 (38%)	1007 (39%)	855 (40%)	1562 (42%)	758 (41%)
2	945 (38%)	1007 (39%)	809 (38%)	1452 (39%)	723 (39%)
3	306 (12%)	330 (13%)	288 (14%)	425 (11%)	224 (12%)
4	177 (7%)	146 (6%)	96 (5%)	163 (4%)	73 (4%)
Missing	119 (5%)	118 (5%)	70 (3%)	120 (3%)	57 (3%)
Grade: 1	296 (12%)	280 (11%)	233 (11%)	435 (12%)	227 (12%)
2	1140 (46%)	1220 (47%)	1016 (48%)	1777 (48%)	897 (49%)
3	898 (36%)	989 (38%)	795 (38%)	1357 (36%)	656 (36%)
Missing	149 (6%)	119 (5%)	74 (3%)	153 (4%)	55 (3%)
Pathway: screen detected	669 (27%)	766 (29%)	625 (30%)	1189 (32%)	547 (30%)
Red flag referral	1385 (56%)	1390 (53%)	1140 (54%)	1803 (48%)	826 (45%)
Death cert only	0–10^a^ (0–0.4%)	0–10^a^ (0–0.4%)	0–10^a^ (0–0.5%)	0–10^a^ (0.0.3%)	0–10^a^ (0–0.5%)
Other	419–429^a^ (17%)	442–452^a^ (17%)	343–353^a^ (16–17%)	720–730^a^ (26–27%)	452–462^a^ (25%)
Mastectomy	669 (27%)	732 (28%)	551 (26%)	960 (26%)	421 (23%)
Breast-conservation	1305 (53%)	1392 (53%)	1230 (58%)	2060 (55%)	1008 (55%)
Radiotherapy	1654 (67%)	1851 (71%)	1567 (74%)	2764 (74%)	1427 (78%)
Systemic therapy	799 (32%)	950 (36%)	814 (38%)	1425 (38%)	763 (42%)
Tamoxifen^b^	831 (33%)	994 (38%)	819 (39%)	1461 (39%)	797 (43%)
Aromatase inhibitors^b^	1649 (66%)	1680 (64%)	1340 (63%)	2381 (64%)	1151 (63%)

^a^Range given to avoid disclosure of small counts; ^b^Medication use at anytime after breast cancer diagnosis

### Breast cancer survival

Association between house value, deprivation and mortality are shown in Table 2 and Supplementary Fig. 1. Based upon the Kaplan–Meier curves (Supplementary Fig. 1), breast cancer survival at 5 years was 83.8% in patients in the least valuable property category compared with 91.1% in patients in the most valuable category. The rate of breast cancer-specific mortality in patients in the least valuable category was 1.90 (95% CI 1.59, 2.27) times that of the most valuable category, and there was a trend across house values (HR per 20 percentile decrease = 1.15 95% CI 1.11, 1.19). This association was slightly attenuated after adjusting for age and comorbidities (adjusted HR = 1.60 95% CI 1.34, 1.92; adjusted HR per 20 percentile decrease = 1.12 95% CI 1.08, 1.16) but further adjustment for deprivation had little impact (adjusted HR = 1.63 95% CI 1.34, 1.99; adjusted HR per 20 percentile decrease = 1.12 95% CI 1.08, 1.17). The association remained after adjusting for stage and grade (adjusted HR = 1.50 95% 1.20, 1.86; adjusted HR per 20 percentile decrease = 1.08 95% CI 1.04, 1.13).

**Table 2:** Association between house value, deprivation and survival

	Nos	Deaths	Person years	Unadjusted HR (95% CI)	P	Adjusted^1^ HR (95% CI)	P	Adjusted for deprivation & house value^2^ HR (95% CI)	P	Additionally adjusted for stage & grade^3^ HR (95% CI)	P
Breast cancer-specific mortality											
House value (per £1000)											
< 75 (lowest)	2483	399	12,927	1.90 (1.59, 2.27)	< 0.001	1.60 (1.34, 1.92)	< 0.001	1.63 (1.34, 1.99)	< 0.001	1.50 (1.20, 1.86)	< 0.001
75–99	2608	352	14,194	1.53 (1.28, 1.84)	< 0.001	1.39 (1.16, 1.67)	< 0.001	1.42 (1.17, 1.72)	< 0.001	1.35 (1.09, 1.66)	0.006
100–124	2118	245	11,818	1.28 (1.05, 1.56)	0.013	1.22 (1.00, 1.48)	0.047	1.24 (1.01, 1.51)	0.037	1.27 (1.02, 1.58)	0.029
125–199	3722	420	21,045	1.24 (1.04, 1.48)	0.018	1.15 (0.96, 1.37)	0.126	1.16 (0.97, 1.39)	0.106	1.24 (1.02, 1.51)	0.03
> 200 (highest)	1835	171	10,638	1.00 (ref cat)		1.00 (ref cat)		1.00 (ref cat)		1.00 (ref cat)	
*Per 20 percentile decrease*				1.15 (1.11, 1.19)	< 0.001	1.12 (1.08, 1.16)	< 0.001	1.12 (1.08, 1.17)	< 0.001	1.08 (1.04, 1.13)	< 0.001
Deprivation											
1 st fifth (deprived)	2151	306	11,574	1.25 (1.07, 1.46)	0.004	1.26 (1.08, 1.47)	0.004	1.00 (0.84, 1.19)	0.987	1.11 (0.91, 1.35)	0.303
2nd fifth	2539	314	13,728	1.09 (0.93, 1.27)	0.297	1.07 (0.92, 1.25)	0.372	0.92 (0.78, 1.09)	0.336	0.92 (0.76, 1.10)	0.357
3rd fifth	2503	297	13,872	1.02 (0.87, 1.20)	0.776	1.04 (0.89, 1.21)	0.647	0.92 (0.78, 1.08)	0.311	0.97 (0.81, 1.16)	0.739
4th fifth	2780	338	15,505	1.05 (0.90, 1.22)	0.557	1.05 (0.90, 1.22)	0.514	0.99 (0.85, 1.15)	0.876	1.02 (0.86, 1.21)	0.826
5th fifth (affluent)	2793	332	15,942	1.00 (ref cat)		1.00 (ref cat)		1.00 (ref cat)		1.00 (ref cat)	
*Per 20 percentile decrease*				1.05 (1.01, 1.08)	0.008	1.05 (1.01, 1.08)	0.011	0.99 (0.95, 1.03)	0.703	1.01 (0.96, 1.05)	0.821
All-cause mortality											
House value (per £1000)											
< 75 (lowest)	2483	746	12,927	2.18 (1.90, 2.50)	< 0.001	1.68 (1.46, 1.92)	< 0.001	1.65 (1.42, 1.92)	< 0.001	1.64 (1.39, 1.94)	< 0.001
75–99	2608	661	14,194	1.76 (1.53, 2.02)	< 0.001	1.50 (1.31, 1.73)	< 0.001	1.49 (1.29, 1.73)	< 0.001	1.50 (1.28, 1.77)	< 0.001
100–124	2118	451	11,818	1.44 (1.24, 1.67)	< 0.001	1.32 (1.14, 1.53)	< 0.001	1.32 (1.13, 1.53)	< 0.001	1.36 (1.15, 1.61)	< 0.001
125–199	3722	748	21,045	1.34 (1.17, 1.54)	< 0.001	1.18 (1.03, 1.36)	0.017	1.18 (1.03, 1.36)	0.017	1.31 (1.13, 1.53)	< 0.001
> 200 (highest)	1835	281	10,638	1.00 (ref cat)		1.00 (ref cat)		1.00 (ref cat)		1.00 (ref cat)	
*Per 20 percentile decrease*				1.19 (1.15, 1.22)	< 0.001	1.13 (1.10, 1.16)	< 0.001	1.12 (1.09, 1.16)	< 0.001	1.10 (1.07, 1.14)	< 0.001
Deprivation											
1 st fifth (deprived)	2151	564	11,574	1.34 (1.19, 1.50)	< 0.001	1.36 (1.21, 1.53)	< 0.001	1.08 (0.95, 1.23)	0.246	1.23 (1.06, 1.42)	0.005
2nd fifth	2539	595	13,728	1.19 (1.06, 1.33)	0.003	1.16 (1.03, 1.30)	0.011	0.99 (0.87, 1.12)	0.847	0.99 (0.86, 1.13)	0.859
3rd fifth	2503	536	13,872	1.06 (0.94, 1.19)	0.313	1.08 (0.96, 1.21)	0.225	0.94 (0.83, 1.07)	0.341	1.00 (0.87, 1.14)	0.959
4th fifth	2780	613	15,505	1.09 (0.97, 1.22)	0.141	1.11 (0.99, 1.25)	0.064	1.04 (0.93, 1.17)	0.515	1.06 (0.93, 1.21)	0.358
5th fifth (affluent)	2793	579	15,942	1.00 (ref cat)		1.00 (ref cat)		1.00 (ref cat)		1.00 (ref cat)	
*Per 20 percentile decrease*				1.07 (1.04, 1.09)	< 0.001	1.07 (1.04, 1.09)	< 0.001	1.01 (0.98, 1.04)	0.547	1.03 (1.00, 1.06)	0.075

^1^Model contains age (in years), year of diagnosis (in years) and comorbidities (myocardial infarction, congestive heart disease, peripheral vascular disease, cerebrovascular disease, chronic pulmonary disease, dementia, liver disease, peptic ulcer disease, diabetes and chronic kidney disease). ^2^Model same as ^1^ plus deprivation and house value. ^3^Model same as ^1^ plus stage, grade, deprivation and house value (complete case *n* = 11,974)

Based upon the Kaplan–Meier curves, breast cancer survival at 5 years was 85.2% in patients in the most deprived areas compared with 87.9% in the least deprived areas. The rate of breast cancer-specific mortality in patients in the most deprived areas was 1.25 (95% CI 1.07, 1.46) times that of the least deprived areas, and there was a weak trend across deprivation levels (HR per 20 percentile decrease = 1.05 95% CI 1.01, 1.08). This association was similar after adjusting for age and comorbidities (adjusted HR = 1.26 95% CI 1.08, 1.47; adjusted HR per 20 percentile decrease = 1.05 95% CI 1.01, 1.08) but was attenuated after further adjustment for house value (adjusted HR = 1.00 95% CI 0.84, 1.19; adjusted HR per 20 percentile decrease = 0.99 95% CI 0.95, 1.03) and stage and grade (adjusted HR = 1.11 95% CI 0.91, 1.35; adjusted HR per 20 percentile decrease = 1.01 95% CI 0.96, 1.05).

### Overall survival

Similar trends were observed for all-cause mortality, with stronger associations for house value than area deprivation. Patients in the lowest house value category had 2.18 times the mortality rate of the highest category (95% CI 1.90, 2.50), attenuating to 1.68 (95% CI 1.46, 1.92) after adjustment for age, year and comorbidities, with minimal further change after adjusting for deprivation and tumour characteristics. Conversely, the most deprived areas showed 1.34 times higher mortality (95% CI 1.19, 1.50) than the least deprived, but this association was substantially attenuated after adjusting for house value (HR 1.08, 95% CI 0.95, 1.23).

### Stage of disease at diagnosis

The association between house value, deprivation and stage at diagnosis is shown in Table 3. Overall, there was evidence of later-stage diagnosis in the least valuable properties, with 7.5% of patients in the least valuable house category diagnosed with stage 4 breast cancer compared with 4.1% of patients in the most valuable category (OR = 1.89 95% CI 1.43, 2.50) and there was a clear trend across house values (OR per 20 percentile decrease = 1.17 95% CI 1.11, 1.24). This association persisted after adjusting for age, year of diagnosis and comorbidities (adjusted OR = 1.65 95% CI 1.24, 2.20) and for deprivation (adjusted OR = 1.70 95% CI 1.24, 2.32). Similarly, 5.9% of patients from the most deprived areas had stage 4 breast cancer compared with 5.0% of patients from the least deprived areas (OR = 1.20 95% CI 0.93, 1.54) and this association had only a weak trend across deprivation levels (OR per 20 percentile decrease = 1.05 95% CI 1.00, 1.11). This association was similar after adjustments for age, year of diagnosis and comorbidities (adjusted OR = 1.21 95% CI 0.94, 1.57) but was completely attenuated after adjusting for house value (adjusted OR = 0.92 95% CI 0.69, 1.23). The associations for house value and deprivation were similar when stage 4 or stage unknown, were compared with stage 1 to 3 (see Table 3). The associations for house value were less marked, but still apparent, when stage 3 and 4 were compared with stage 1 and 2 (Supplementary Table 1).

**Table 3:** Association between house value and stage at breast cancer diagnosis

	Proportion stage 4 (n/N)	Unadjusted OR (95% CI)	P	Adjusted^1^ OR (95% CI)	P	Adjusted for deprivation & house value^2^ OR (95% CI)	P
Stage 4 versus stage 1 to 3 at diagnosis							
House value (per £1000)							
< 75 (lowest)	7.5% (177/2364)	1.89 (1.43, 2.50)	< 0.001	1.65 (1.24, 2.20)	0.001	1.70 (1.24, 2.32)	0.001
75–99	5.9% (146/2490)	1.45 (1.09, 1.94)	0.011	1.36 (1.02, 1.82)	0.038	1.39 (1.02, 1.90)	0.035
100–124	4.7% (96/2048)	1.15 (0.84, 1.57)	0.383	1.09 (0.79, 1.49)	0.612	1.10 (0.80, 1.52)	0.556
125–199	4.5% (163/3602)	1.11 (0.83, 1.47)	0.48	1.06 (0.79, 1.40)	0.713	1.06 (0.80, 1.42)	0.672
> 200 (highest)	4.1% (73/1778)	1.00 (ref cat)		1.00 (ref cat)		1.00 (ref cat)	
*Per 20 percentile decrease*		1.17 (1.11, 1.24)	< 0.001	1.14 (1.07, 1.21)	< 0.001	1.14 (1.07, 1.22)	< 0.001
Deprivation							
1 st fifth (deprived)	5.9% (123/2071)	1.20 (0.93, 1.54)	0.157	1.21 (0.94, 1.57)	0.137	0.92 (0.69, 1.23)	0.564
2nd fifth	5.8% (141/2448)	1.16 (0.91, 1.48)	0.228	1.16 (0.91, 1.49)	0.228	0.97 (0.75, 1.27)	0.838
3rd fifth	5.1% (123/2395)	1.03 (0.80, 1.32)	0.828	1.07 (0.83, 1.37)	0.626	0.93 (0.71, 1.21)	0.579
4th fifth	5.0% (133/2669)	1.00 (0.78, 1.27)	0.975	1.00 (0.78, 1.28)	0.996	0.93 (0.73, 1.20)	0.602
5th fifth (affluent)	5.0% (135/2699)	1.00 (ref cat)		1.00 (ref cat)		1.00 (ref cat)	
*Per 20 percentile decrease*		1.05 (1.00, 1.11)	0.074	1.05 (1.00, 1.12)	0.065	0.99 (0.93, 1.06)	0.772
Stage 4 or stage unknown versus stage 1 to 3 at diagnosis							
House value (per £1000)							
< 75 (lowest)	11.9% (296/2483)	1.78 (1.43, 2.20)	< 0.001	1.34 (1.07, 1.67)	0.011	1.35 (1.05, 1.72)	0.018
75–99	10.1% (264/2608)	1.48 (1.19, 1.84)	< 0.001	1.24 (0.99, 1.55)	0.064	1.24 (0.98, 1.58)	0.077
100–124	7.8% (166/2118)	1.12 (0.88, 1.42)	0.37	0.99 (0.77, 1.27)	0.937	0.99 (0.77, 1.27)	0.928
125–199	7.6% (283/3722)	1.08 (0.87, 1.34)	0.488	0.95 (0.76, 1.18)	0.621	0.94 (0.75, 1.18)	0.609
> 200 (highest)	7.1% (130/1835)	1.00 (ref cat)		1.00 (ref cat)		1.00 (ref cat)	
*Per 20 percentile decrease*		1.16 (1.11, 1.21)	< 0.001	1.10 (1.05, 1.15)	< 0.001	1.10 (1.04, 1.16)	< 0.001
Deprivation							
1 st fifth (deprived)	9.4% (203/2151)	1.17 (0.96, 1.42)	0.127	1.20 (0.98, 1.47)	0.085	0.99 (0.78, 1.24)	0.912
2nd fifth	9.1% (232/2539)	1.13 (0.93, 1.36)	0.224	1.13 (0.93, 1.38)	0.216	1.00 (0.81, 1.24)	0.99
3rd fifth	9.2% (231/2503)	1.14 (0.94, 1.38)	0.184	1.16 (0.95, 1.41)	0.147	1.05 (0.85, 1.29)	0.637
4th fifth	8.8% (244/2780)	1.08 (0.89, 1.30)	0.439	1.09 (0.90, 1.32)	0.395	1.04 (0.85, 1.27)	0.694
5th fifth (affluent)	8.2% (229/2793)	1.00 (ref cat)		1.00 (ref cat)		1.00 (ref cat)	
*Per 20 percentile decrease*		1.04 (0.99, 1.08)	0.106	1.04 (1.00, 1.09)	0.078	1.00 (0.95, 1.05)	0.876

^1^Model contains age (in years), year of diagnosis (in years) and comorbidities (myocardial infarction, congestive heart disease, peripheral vascular disease, cerebrovascular disease, chronic pulmonary disease, dementia, liver disease, peptic ulcer disease, diabetes and chronic kidney disease). ^2^Model same as ^1^ plus deprivation and house value

Sensitivity analyses using missing indicator category or multiple imputation for missing data (Table 4) were generally similar to main findings, however, individuals with missing house valuations had substantially higher breast cancer-specific mortality (HR = 2.93 95% CI 2.40, 3.57).

**Table 4:** Sensitivity analyses for the association between house value and breast cancer survival

Outcome	Nos	Breast cancer deaths	Person Years	Unadjusted HR (95% CI)	P	Adjusted^1^ HR (95% CI)	P	Adjusted for deprivation & house value^2^ HR (95% CI)	P
A. Including missing house value and deprivation as separate category									
House value (per £1000)									
< 75 (lowest)	2504	403	13,036	1.88 (1.57, 2.25)	< 0.001	1.56 (1.31, 1.87)	< 0.001	1.58 (1.30, 1.92)	< 0.001
75–99	2630	354	14,329	1.51 (1.26, 1.81)	< 0.001	1.36 (1.14, 1.64)	0.001	1.39 (1.15, 1.68)	0.001
100–124	2136	247	11,899	1.27 (1.05, 1.54)	0.015	1.20 (0.99, 1.46)	0.063	1.22 (1.00, 1.49)	0.049
125–199	3748	421	21,207	1.22 (1.02, 1.46)	0.027	1.13 (0.94, 1.35)	0.185	1.14 (0.95, 1.36)	0.155
> 200 (highest)	1852	174	10,738	1.00 (ref cat)		1.00 (ref cat)		1.00 (ref cat)	
Missing	976	221	4608	2.93 (2.40, 3.57)	< 0.001	2.25 (1.84, 2.76)	< 0.001	2.31 (1.88, 2.85)	< 0.001
Deprivation									
1 st fifth (deprived)	2298	342	12,285	1.22 (1.05, 1.41)	0.008	1.24 (1.07, 1.43)	0.005	1.00 (0.85, 1.18)	0.978
2nd fifth	2741	360	14,733	1.07 (0.93, 1.24)	0.35	1.07 (0.93, 1.24)	0.359	0.92 (0.79, 1.07)	0.296
3rd fifth	2705	343	14,931	1.02 (0.88, 1.18)	0.823	1.04 (0.90, 1.20)	0.624	0.91 (0.78, 1.06)	0.243
4th fifth	2979	377	16,428	1.02 (0.88, 1.17)	0.819	1.03 (0.89, 1.19)	0.679	0.96 (0.83, 1.11)	0.562
5th fifth (affluent)	2938	371	16,433	1.00 (ref cat)		1.00 (ref cat)		1.00 (ref cat)	
Missing	185	27	1006	1.20 (0.81, 1.78)	0.351	1.23 (0.83, 1.82)	0.303	0.84 (0.56, 1.25)	0.393
B. Using multiple imputation to impute house value and deprivation^3^									
House value (per £1000)									
< 75 (lowest)	2731	469	14,048	1.90 (1.60, 2.25)	< 0.001	1.58 (1.33, 1.87)	< 0.001	1.61 (1.33, 1.95)	< 0.001
75–99	2850	404	15,353	1.54 (1.30, 1.83)	< 0.001	1.39 (1.17, 1.65)	< 0.001	1.42 (1.18, 1.70)	< 0.001
100–124	2276	282	12,588	1.29 (1.06, 1.57)	0.01	1.22 (1.00, 1.49)	0.049	1.24 (1.01, 1.52)	0.036
125–199	4019	471	22,529	1.21 (1.02, 1.45)	0.031	1.12 (0.94, 1.34)	0.202	1.13 (0.95, 1.35)	0.167
> 200 (highest)	1970	194	11,299	1.00 (ref cat)		1.00 (ref cat)		1.00 (ref cat)	
Deprivation									
1 st fifth (deprived)	2332	347	12,455	1.22 (1.05, 1.41)	0.008	1.23 (1.06, 1.43)	0.005	0.98 (0.83, 1.16)	0.832
2nd fifth	2772	368	14,882	1.07 (0.93, 1.24)	0.341	1.07 (0.93, 1.24)	0.355	0.92 (0.79, 1.07)	0.286
3rd fifth	2741	347	15,133	1.01 (0.88, 1.17)	0.85	1.03 (0.89, 1.20)	0.652	0.92 (0.79, 1.07)	0.267
4th fifth	3024	380	16,671	1.01 (0.88, 1.17)	0.873	1.02 (0.89, 1.18)	0.74	0.96 (0.83, 1.11)	0.598
5th fifth (affluent)	2977	378	16,677	1.00 (ref cat)		1.00 (ref cat)		1.00 (ref cat)	

^1^Model contains age (in years), year of diagnosis (in years) and comorbidities (myocardial infarction, congestive heart disease, peripheral vascular disease, cerebrovascular disease, chronic pulmonary disease, dementia, liver disease, peptic ulcer disease, diabetes and chronic kidney disease). ^2^Model same as ^1^ plus deprivation and house value. ^3^Number of women, breast cancer deaths and person years based upon first imputed dataset

## Discussion

In this population-based study of 12,766 women diagnosed with breast cancer in NI, low house value and high deprivation were both associated with lower breast cancer survival and more frequent stage 4 disease. However, the observed associations were more pronounced for house value, an individual-level measure of SEP, than the more widely used area-based NIMDM. For example, women in the lowest valued properties had a 60% increase in breast cancer-specific mortality compared with women in the highest valued properties, and this association persisted after further adjustment for deprivation. In contrast, women in the most deprived areas had a 26% increase in breast cancer-specific mortality compared with women in the least deprived areas, with the association substantially attenuated after adjustment for house value, suggesting that individual-level SEP measures capture a different aspect of socioeconomic influences on breast cancer outcomes than area-based measures.

Our study’s findings of poorer breast cancer survival among women with lower SEP are consistent with previous research demonstrating socioeconomic gradients in breast cancer outcomes [5, 34–36]. Few studies have specifically examined house value in relation to breast cancer survival, but available evidence supports our findings. A Singaporean study observed a 63% increase in cancer-specific mortality in women living in the lowest versus highest property value categories, though this was attenuated to 14% after adjusting for confounders including tumour stage [22]. Similarly, a USA study demonstrated reduced breast cancer-specific mortality with increasing house value [23]. Our study observed a 60% increase in mortality for the lowest versus highest house value categories, which remained substantial even after adjustment for tumour characteristics.

Previous studies using other individual measures of SEP, such as income, occupation, and education, have generally demonstrated poorer breast cancer survival among lower SEP groups. A systematic review found that occupation, income, and education were associated with poorer breast cancer survival [6], though none of the included studies were UK-based. A previous UK study investigating census-based individual-level SEP measures showed limited evidence of associations between education or occupation and 5-year net survival but found improved survival among individuals with the highest income estimated from occupation [5]. Similarly, a Finnish study reported 19% higher breast cancer mortality risk in women with lower education [14]. These patterns extend to overall survival, with a European systematic review reporting poorer overall survival associated with lower education and occupation, and a US study reporting poorer survival in women with lower income [15, 16].

Direct comparison with these studies is challenging as we used house value as our individual-level SEP measure. The strong socioeconomic gradient we observed could reflect the high levels of deprivation in NI [28, 37] and/or the long waiting lists for healthcare, which may exacerbate inequalities if more affluent women can access private healthcare to overcome delays. Data on private healthcare utilisation were not available precluding further investigation of this mechanism.

Our findings also suggest that the individual-level SEP indicators may be more sensitive than area-level measures for detecting socioeconomic differences in breast cancer survival. Area-based analyses may dilute true SEP effects because all individuals within a specific area are assigned the same average deprivation level regardless of their household circumstances [12]. This limitation may be particularly relevant for the NIMDM which uses Super Output Areas which averages approximately 2100 people, potentially masking individual-level socioeconomic variation.

The mechanisms underlying the observed socioeconomic inequalities are likely multifactorial. One explanation may be the differences in rates of breast cancer screening, with those more socially disadvantaged less likely to attend breast screening and more likely to be diagnosed with advanced disease and have poorer outcomes [38, 39]. While NI has substantially less ethnic diversity than other UK regions, it exhibits higher levels of social deprivation [37, 40]. Previous studies suggest that patients from lower SEP may have different tumour characteristics, including later-stage disease [41, 42]. Indeed, we observed evidence of later-stage disease presentation among patients with lower house values, although associations with mortality persisted after adjustments for stage, suggesting additional pathways beyond diagnostic timing. Furthermore, patients from lower SEP backgrounds may experience healthcare differently, including altered referral patterns and treatment provision [41].

Patients from lower SEP may experience poorer health and more behavioural risk factors, including weight, diet, alcohol intake and exercise, which could impact cancer outcomes. However, we did not capture any data on lifestyle characteristics. Furthermore, lower health literacy, particularly prevalent among deprived populations, may limit breast health awareness and symptom recognition, potentially contributing to diagnostic delays and reduced screening [4]. Further research investigating healthcare, lifestyle factors and other individual-level SEP measures in NI (such as education, occupation, transport access and disability status) is needed to better understand the mechanisms underlying the association between house value and breast cancer outcomes. Additionally, as we investigated only breast cancer, which has a widely publicised screening program, well-known risk factors, and established treatment pathways, future research should determine whether house value indicates health inequalities for other cancer sites.

### Strengths and limitations

This study has several strengths. First, we utilised a large, well-characterised cohort from the NICR with an estimated 99% completeness rate. Second, we had comprehensive coverage of domestic property valuations [17] and complete follow-up for survival outcomes minimising loss to follow-up bias. Third, we employed rigorous statistical methods, including multiple imputation and appropriate confounder adjustment.

Our study has various limitations. House value data were missing for 7% of patients because the address did not match a UPRN, potentially reflecting incorrect addresses or properties without domestic valuations (e.g. residential care settings, those with no fixed address), which could explain the higher mortality observed in patients with missing house values. The house values our analysis were used to allocate local tax in 2025, but they are based upon what the house could have reasonably sold for in 2005 and over the last two decades absolute house prices have considerably changed. However, this approach should not introduce bias provided the relative socioeconomic ranking of property values across different areas remained stable over time, even when absolute property values change, as the measure captures relative rather than absolute wealth position within a population. House value may not adequately reflect the resident’s current financial position as their wealth may be tied up in assets, but they have limited disposable income. Additionally, we lacked data on home ownership. While over 70% of homes among persons aged 65 years and older in NI are owner-occupied [17], house value may still serve as a reasonable SEP marker for private renters, and public sector tenants are likely to rent properties in lower-value categories. Nevertheless, exposure misclassification may occur between tenants and homeowners in similar-value properties due to wealth differences. We could not adjust for clustering as we only had access to the NIMDM quintile and not the Super Output Area of the patient’s address. This may have led to an overestimation of statistical power and potentially inflated the precision of our estimates related to deprivation as individuals within the same area may share correlated health outcomes. However, this should not impact upon the individual-level analysis of house value.

In summary, house value demonstrated stronger associations with stage 4 disease and breast cancer survival than area-level deprivation measures, indicating that individual-level SEP indicators are more sensitive for detecting socioeconomic health inequalities. Our findings establish that publicly available property valuations provide a feasible approach for implementing such individual-level surveillance at the population level. Further research is warranted to better understand the mechanisms underlying these inequalities and to inform targeted interventions aimed at reducing socioeconomic disparities in breast cancer outcomes.

## Supplementary Information

Below is the link to the electronic supplementary material.

Supplementary file1 (DOCX 234 KB)

## Data Availability

The dataset can be recreated by authorised researchers, at a cost, after obtaining the necessary ethical approvals and applying to the Northern Ireland Cancer Registry. Further information on how to apply can be found here: (https://www.qub.ac.uk/research-centres/nicr/CancerInformation/requests). Analysis codes are available upon request to the corresponding author.
