# Supplementary material for: House value as an individual socioeconomic indicator for breast cancer survival and late-stage diagnosis: a population-based cohort study from Northern Ireland
Source: Breast Cancer Res Treat. 2026 Mar 21;216(3):33. doi: 10.1007/s10549-026-07947-z (PMC13005866; doi:10.1007/s10549-026-07947-z)
Supplement: Supplementary file 1 — Supplementary file1 (DOCX 234 KB) [file 10549_2026_7947_MOESM1_ESM.docx]

**House value as an individual socioeconomic indicator for breast cancer survival and late-stage diagnosis: a population-based cohort study from Northern Ireland**

**Supplementary Materials**

Supplementary Figure 1. Kaplan-Meier survival curves for breast cancer-specific and all-cause mortality by house value and deprivation.

**Key**

House value category (in £1000s) █ <75 [lowest] █ 75-99 █ 100-124 █ 125-199 █ 200 [highest]

Deprivation fifth █ 1^st^ [deprived] █ 2^nd^ █ 3^rd^ █ 4^th^ █ 5^th^ [affluent]

Supplementary Table 1 Association between house value, deprivation and proportion late stage considering late stage 3 & 4

| Outcome | Proportion late stage (n/N) | Unadjusted  OR (95% CI) | P | Adjusted^1^ OR  (95% CI) | P | Adjusted (+hv/dep)^2^  OR (95% CI) | P |
| --- | --- | --- | --- | --- | --- | --- | --- |
| Late stage (Stage 3 & 4 versus Stage1 & 2) | | | | | | | |
| House value |  |  |  |  |  |  |  |
| <75 | 20.4% (483/2364) | 1.28 (1.09, 1.50) | 0.002 | 1.24 (1.05, 1.45) | 0.01 | 1.23 (1.03, 1.47) | 0.02 |
| 75-100 | 19.1% (476/2490) | 1.18 (1.00, 1.38) | 0.044 | 1.16 (0.99, 1.36) | 0.074 | 1.16 (0.98, 1.37) | 0.093 |
| 100-125 | 18.8% (384/2048) | 1.15 (0.97, 1.36) | 0.099 | 1.14 (0.96, 1.35) | 0.124 | 1.14 (0.96, 1.35) | 0.136 |
| 125-200 | 16.3% (588/3602) | 0.97 (0.84, 1.13) | 0.724 | 0.96 (0.83, 1.12) | 0.64 | 0.96 (0.83, 1.12) | 0.633 |
| >200 | 16.7% (297/1778) | 1.00 (ref cat) | . | 1.00 (ref cat) | . | 1.00 (ref cat) | . |
| Per 20% increase |  | 1.07 (1.04, 1.11) | <0.001 | 1.07 (1.03, 1.10) | <0.001 | 1.06 (1.03, 1.10) | 0.001 |
| Deprivation |  |  |  |  |  |  |  |
| 1^st^ fifth (deprived) | 18.9% (392/2071) | 1.12 (0.96, 1.29) | 0.148 | 1.12 (0.96, 1.30) | 0.145 | 0.99 (0.83, 1.16) | 0.865 |
| 2^nd^ fifth | 19.6% (481/2448) | 1.17 (1.02, 1.35) | 0.03 | 1.18 (1.02, 1.36) | 0.025 | 1.08 (0.93, 1.26) | 0.288 |
| 3^rd^ fifth | 16.9% (404/2395) | 0.97 (0.84, 1.12) | 0.681 | 0.98 (0.85, 1.14) | 0.823 | 0.93 (0.79, 1.08) | 0.315 |
| 4^th^ fifth | 18.1% (484/2669) | 1.06 (0.92, 1.22) | 0.425 | 1.06 (0.92, 1.22) | 0.4 | 1.03 (0.89, 1.19) | 0.684 |
| 5^th^ fifth (affluent) | 17.3% (467/2699) | 1.00 (ref cat) | . | 1.00 (ref cat) | . | 1.00 (ref cat) | . |
| Per 20% increase |  | 1.03 (1.00, 1.07) | 0.055 | 1.03 (1.00, 1.07) | 0.05 | 1.00 (0.97, 1.04) | 0.854 |
| Late stage (3 & 4 & unknown stage versus 1 & 2) | | | | | | | |
| House value |  |  |  |  |  |  |  |
| <75 | 24.2% (602/2483) | 1.34 (1.15, 1.55) | <0.001 | 1.20 (1.03, 1.39) | 0.018 | 1.19 (1.01, 1.41) | 0.035 |
| 75-100 | 22.8% (594/2608) | 1.23 (1.06, 1.43) | 0.005 | 1.16 (1.00, 1.34) | 0.058 | 1.15 (0.98, 1.35) | 0.082 |
| 100-125 | 21.4% (454/2118) | 1.14 (0.98, 1.33) | 0.096 | 1.10 (0.94, 1.29) | 0.234 | 1.09 (0.93, 1.28) | 0.274 |
| 125-200 | 19.0% (708/3722) | 0.98 (0.85, 1.13) | 0.81 | 0.94 (0.81, 1.08) | 0.373 | 0.93 (0.81, 1.08) | 0.351 |
| >200 | 19.3% (354/1835) | 1.00 (ref cat) | . | 1.00 (ref cat) | . | 1.00 (ref cat) | . |
| Per 20% increase |  | 1.09 (1.06, 1.12) | <0.001 | 1.06 (1.03, 1.10) | <0.001 | 1.06 (1.03, 1.10) | 0.001 |
| Deprivation |  |  |  |  |  |  |  |
| 1^st^ fifth (deprived) | 21.9% (472/2151) | 1.12 (0.97, 1.28) | 0.111 | 1.13 (0.98, 1.30) | 0.083 | 1.00 (0.86, 1.17) | 0.985 |
| 2^nd^ fifth | 22.5% (572/2539) | 1.16 (1.01, 1.32) | 0.03 | 1.17 (1.02, 1.34) | 0.021 | 1.08 (0.94, 1.24) | 0.278 |
| 3^rd^ fifth | 20.5% (512/2503) | 1.02 (0.89, 1.17) | 0.738 | 1.03 (0.90, 1.19) | 0.622 | 0.98 (0.85, 1.12) | 0.727 |
| 4^th^ fifth | 21.4% (595/2780) | 1.08 (0.95, 1.23) | 0.225 | 1.09 (0.96, 1.24) | 0.192 | 1.06 (0.93, 1.21) | 0.395 |
| 5^th^ fifth (affluent) | 20.1% (561/2793) | 1.00 (ref cat) | . | 1.00 (ref cat) | . | 1.00 (ref cat) | . |
| Per 20% increase |  | 1.03 (1.00, 1.06) | 0.056 | 1.03 (1.00, 1.06) | 0.038 | 1.00 (0.97, 1.04) | 0.821 |

^1^Model contains age (in years), year of diagnosis (in years) and comorbidities (MI, CHF, PVD, stroke, COPD, dementia, liver, peptic ulcer disease, diabetes and CKD). ^2^Model same as ^1^ plus deprivation and house value
